# Cost‐Effectiveness of Radiofrequency Ablation for Benign Non‐Functioning Thyroid Nodules: A Systematic Review

**DOI:** 10.1111/coa.70150

**Published:** 2026-08-02

**Authors:** Sam P. J. van Dijk, Matthew M. K. Blanco, Ali Akhavan, Jessica Liu McMullin, G. Scott Gazelle, Mohammad S. Jalali, Jagpreet Chhatwal, Yinin Hu, Tessa M. van Ginhoven, Rajshri M. Gartland, Carrie Cunningham

**Affiliations:** ^1^ Department of Surgical Oncology and Gastrointestinal Surgery Academic Center for Thyroid Disease, Erasmus MC Cancer Institute Rotterdam the Netherlands; ^2^ Department of Surgery Massachusetts General Hospital, Harvard Medical School Boston Massachusetts USA; ^3^ Sloan School of Management, Massachusetts Institute of Technology Cambridge Massachusetts USA; ^4^ Department of Surgery University of Utah Salt Lake City Utah USA; ^5^ Department of Radiology Massachusetts General Hospital, Harvard Medical School Boston Massachusetts USA; ^6^ MGH Institute for Technology Assessment, Harvard Medical School Boston Massachusetts USA; ^7^ Division of Surgical Oncology, Department of Surgery University of Maryland Baltimore Baltimore Maryland USA

**Keywords:** benign thyroid nodules, cost‐effectiveness, radiofrequency ablation

## Abstract

**Purpose:**

This systematic review tests the hypothesis that radiofrequency ablation (RFA) for benign non‐functioning thyroid nodules is associated with favourable cost‐effectiveness compared with surgery.

**Materials and Methods:**

A systematic literature search of multiple databases (Embase, Medline, Web of Science, and Cochrane Central) was conducted through 16 May 2025, with no restrictions on publication date or language. Full economic evaluations comparing RFA with alternative management strategies were included in the primary synthesis. Secondary narrative synthesis included studies reporting cost data without formal cost‐effectiveness modelling. All costs were converted to 2021 US dollars to enable cross‐study comparability. Incremental cost‐effectiveness ratios (ICERs), net monetary benefit (NMB), and net health benefit (NHB) were calculated where data allowed.

**Results:**

Thirteen studies met the inclusion criteria: 3 full economic evaluations and 10 descriptive cost studies. All compared RFA to surgery. In the primary synthesis, two studies reported ICERs for RFA versus surgery, which ranged from –$14 994 (reflecting a lower cost and greater effectiveness for RFA) to $98 858 per QALY gained, with substantial variation in model design and assumptions. Post hoc estimates of NMB and NHB generally favoured RFA at $50 000 and $100 000 willingness‐to‐pay thresholds. In the narrative synthesis, 8 of 10 studies reported lower total costs for RFA (median: $2499) compared to surgery (median: $3579). Methodological heterogeneity limited comparability. Overall certainty of economic evidence was rated as low.

**Conclusion:**

RFA may be a cost‐effective alternative to surgery for benign thyroid nodules, but current economic evidence is limited and of low certainty.

## Key Points

- RFA offers a potentially cost‐effective, minimally invasive alternative to surgery for benign non‐functioning thyroid nodules.
- Most available studies report lower costs for RFA, largely due to outpatient treatment and avoidance of hospital admission and general anaesthesia.
- The economic value of RFA varies considerably across healthcare systems, procedural costs, and analytical assumptions.
- Current evidence is limited by methodological heterogeneity, short follow‐up, and insufficient incorporation of long‐term costs and quality of life.
- Robust, context‐specific economic evaluations are needed to support reimbursement decisions and wider implementation of RFA.

## 1 Introduction

Benign non‐functioning thyroid nodules are a common clinical finding, detectable by ultrasound in approximately 34%–68% of the general adult population [1, 2]. While the majority of benign non‐functioning nodules remain asymptomatic, approximately 5%–10% may enlarge over time and become clinically relevant, leading to compressive symptoms, cosmetic concerns, or anxiety that may warrant intervention [3]. Traditionally, lobectomy has been the most common treatment for unilateral nodule(s), entailing risks such as hypothyroidism and recurrent laryngeal nerve injury [4]. To meet the demand for less invasive treatment, thermal ablation techniques have emerged over the past two decades as alternatives to surgery for patients with a solitary or dominant benign non‐functioning thyroid nodule.

Radiofrequency ablation (RFA) is the most studied and widely adopted technique, delivering targeted thermal energy to reduce nodule volume while preserving thyroid function [5]. Clinical evidence shows that RFA effectively relieves symptoms with a low complication rate and has been endorsed as a first‐line option in several international guidelines [6, 7, 8, 9, 10]. As healthcare systems increasingly emphasize value‐based care, interventions like RFA must demonstrate not only clinical benefits but also economic justification. While RFA may reduce upfront costs compared to surgery by avoiding hospitalization and general anesthesia, questions remain about its long‐term cost‐effectiveness, particularly considering possible repeat procedures and follow‐up requirements. Unlike in oncology, where early robust cost‐effectiveness data facilitated the uptake of RFA [11], similar high‐quality evaluations are lacking in benign thyroid disease and may hinder broader implementation [12]. To date, no comprehensive synthesis of available economic evidence of RFA in benign thyroid nodules exists.

This systematic review aims to identify, critically appraise, and summarise cost‐effectiveness studies on RFA for benign thyroid nodules to inform clinicians, policymakers, and guideline developers about its economic value and potential role in thyroid care.

## 2 Materials and Methods

### 2.1 Literature Search

This systematic review followed the PRISMA 2020 guidelines and the PRISMA‐S extension for reporting search strategies, and was further guided by the Cochrane Handbook for Systematic Reviews of Interventions (Chapter 20 on economic evidence) and the ISPOR Task Force recommendations for reviews of cost and cost‐effectiveness studies [13, 14, 15, 16]. The study protocol was registered in PROSPERO (CRD‐420251063309). A comprehensive search strategy was developed in Embase.com by an experienced medical librarian and translated to other databases (Medline via Ovid, Web of Science, and Cochrane Central) using Bramer's method [17]. The final search was performed on 16 May 2025, without language or publication date restrictions. Full search strategies are available in Appendix E1.

### 2.2 Eligibility Criteria

Full economic evaluations of RFA for benign, non‐functioning thyroid nodules, comparing RFA to alternatives such as surgery, active surveillance, or other ablation techniques were included. Eligible studies assessed both costs and health outcomes using formal cost‐effectiveness, cost‐utility, cost–benefit, or cost‐minimization analyses [13]. No restrictions were placed on the country, language, study design, setting, or analytical perspective. Both peer‐reviewed journal articles and relevant grey literature (e.g., dissertations or institutional reports) were included if they met the inclusion criteria. Studies reporting cost data without formal full economic evaluations were included in a secondary narrative synthesis. Studies were excluded if they (1) exclusively evaluated other thermal ablation techniques than RFA; (2) focused exclusively on malignant thyroid disease or autonomously functioning thyroid nodules; or (3) were non‐original reports such as abstracts, editorials, reviews, protocols, or expert statements.

### 2.3 Article Selection and Data Extraction

Two reviewers (S.D. and M.B.) independently screened all titles, abstracts, and full texts for eligibility using Covidence, resolving discrepancies by consensus or a third reviewer (C.C.). One reviewer extracted data, which was then independently checked by a second reviewer for completeness and accuracy. A standardized extraction form was used to collect data on study design, population, interventions, comparators, utilities, costs, and cost‐effectiveness outcomes (Appendix E2).

### 2.4 Outcomes

The primary outcome was the incremental cost‐effectiveness ratio (ICER), expressed as cost per quality‐adjusted life‐year (QALY) gained. Secondary outcomes included net health benefit (NHB) and net monetary benefit (NMB), which are alternative ways of expressing cost‐effectiveness. NHB reformulates outcomes in terms of QALYs, while NMB expresses them in monetary terms, thereby allowing results to be more easily compared across different willingness‐to‐pay (WTP) thresholds. In the United States, thresholds of $50 000 to $100 000 per QALY are commonly applied, and these values were used in this review to contextualize the results. Additional secondary outcomes included direct and indirect costs, utility scores, and the methodological quality of the studies.

### 2.5 Quality of Included Studies

Methodological quality was assessed using the Consensus on Health Economic Criteria (CHEC) list for trial‐based evaluations, ECOBIAS for model‐based studies, and the Newcastle‐Ottawa Scale for observational cost comparisons [18, 19, 20]. Quality of studies without clinical outcomes or utility measures was assessed narratively using the CHEERS 2022 criteria [21]. Two reviewers (S.D. and M.B.) independently assessed study quality, with disagreements resolved by consensus or third‐party input (C.C.).

### 2.6 Data Synthesis

Given the anticipated heterogeneity in study designs, perspectives, and outcome measures, no meta‐analysis was performed. To enable cross‐study comparability, all cost values were converted to 2021 US dollars, reflecting the most recent cost data reported in the included studies, using country‐specific inflation indices and World Bank exchange rates (Appendices E3 and E4). For studies lacking a cost year, the midpoint of data collection was used as a proxy. A structured narrative synthesis of ICERs, total and incremental costs, and QALY estimates, stratified by study design, comparator, perspective, and key assumptions was performed. Where data allowed, NMB and NHB were calculated using WTP thresholds of $50 000 and $100 000 per QALY. Studies reporting RFA cost data, but no full economic evaluation, were summarised separately to reflect real‐world expenditures and expose gaps in formal health economic evidence.

### 2.7 Certainty Assessment and Publication Bias

Certainty of the economic evidence was assessed using a modified GRADE approach tailored to economic evaluations [22]. This included structured domain‐level assessments of risk of bias, inconsistency, indirectness, imprecision, and publication bias. The risk of publication and reporting bias was qualitatively evaluated through trial registry checks (e.g., ClinicalTrials.gov, PROSPERO) and grey literature searches.

## 3 Results

### 3.1 Systematic Literature Search and Study Selection

The literature search was conducted on 16 May 2025. A total of 340 articles were identified, of which 206 unique records were screened after duplicate removal. Full‐text assessment was performed for 23 articles, resulting in 13 studies meeting the inclusion criteria (Figure 1). Among these, three studies were included in the primary synthesis (full economic evaluations) [23, 24, 25] and 10 studies contributed to the secondary narrative synthesis (cost comparisons) [26, 27, 28, 29, 30, 31, 32, 33, 34, 35].

**FIGURE 1:**
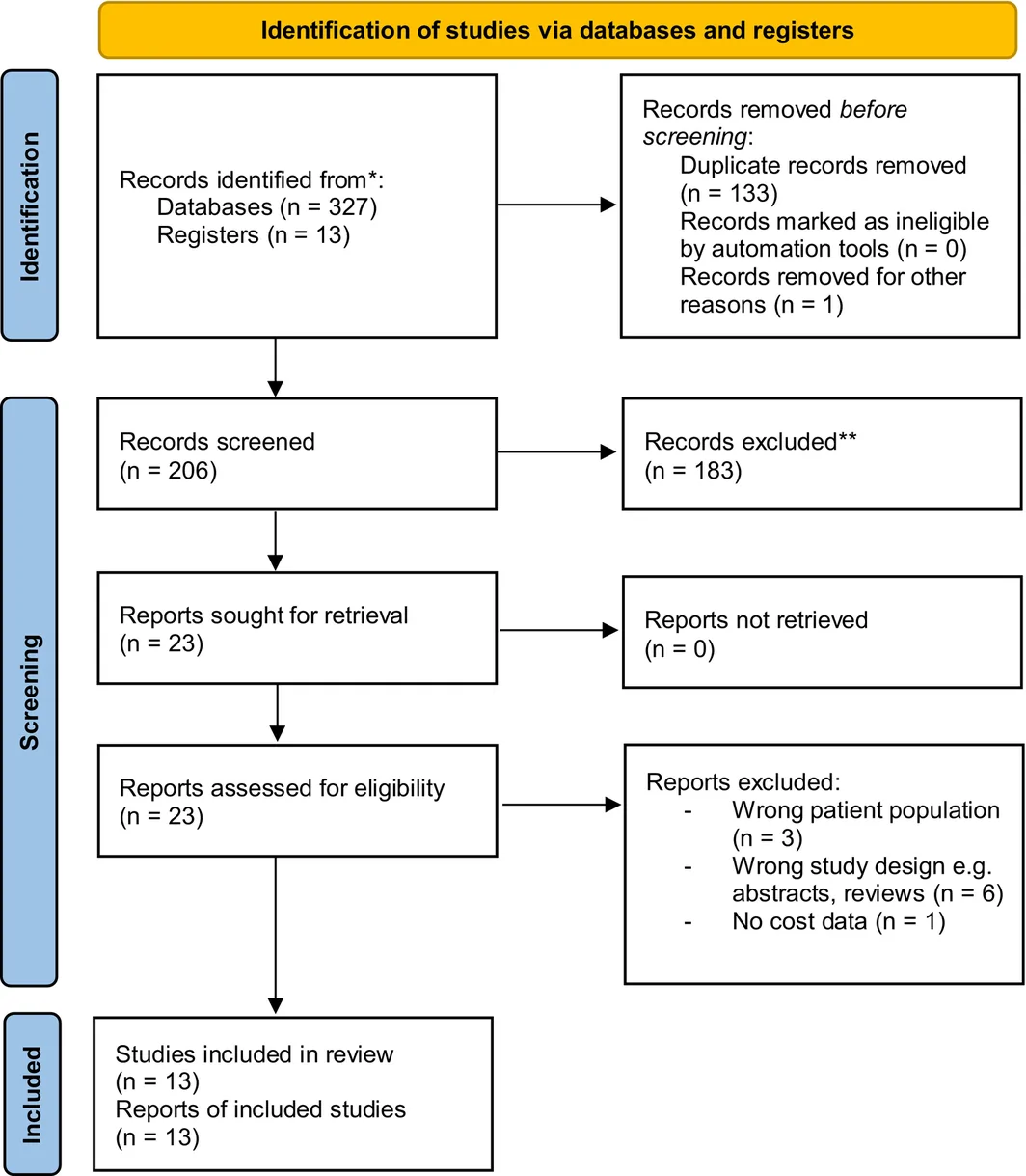
PRISMA 2020 flow diagram of study selection. *Consider, if feasible to do so, reporting the number of records identified from each database or register searched (rather than the total number across all databases/registers). **If automation tools were used, indicate how many records were excluded by a human and how many were excluded by automation tools.

### 3.2 Primary Synthesis

#### 3.2.1 Economic Evaluation Methods, Costs, Utilities, and Methodological Quality

Three studies conducted formal economic evaluations: two cost‐utility analyses and one cost‐minimization analysis. An overview of their characteristics and outcomes is provided in Table 1. Details on methodological appraisal are available in Appendix E5.

**TABLE 1:** Characteristics and outcomes of full economic evaluations.

Study (author, year, country)	Yue et al. (2016, China) [25]	Kuo et al. (2023, USA) [23]	Papi et al. (2023, Italy) [24]
Study design	Propensity scored retrospective cohort study	Patient‐level state transition microsimulation study	Retrospective cohort study
Economic evaluation	Cost Utility Analysis	Cost Utility Analysis	Cost‐Minimization Analysis
Perspective	Societal	Third‐party payer	Societal
Time horizon	6 months	Lifetime	12 months
Discount Rate	Not applied	3% per year	Not applied
Intervention vs. Comparator(s)	RFA vs. total thyroidectomy	RFA vs. lobectomy	RFA vs. lobectomy, total thyroidectomy and LTA
Cost sources	Hospital registries and national guidelines (including medications, surgical materials, and hospital stays); indirect costs estimated using GDP per capita.	Broad cost categories from published literature; no detailed cost breakdown; excluded indirect costs.	Italian tariffs and hospital records; included direct and some indirect costs (productivity loss).
Total Costs (Intervention)	$3369.30	$16 563	$2408.86
Total Costs (Comparator)	$2380.72	$19 262	$4903.40 (lobectomy) $6048.22 (total thyroidectomy) $1694.62 (LTA 1 fibre) $2165.21 (LTA 2 fibres)
Incremental costs (USD)	$988.58	−$2699	−$2494.54 (RFA vs. lobectomy); −$3639.36 (RFA vs. total thyroidectomy) $714.24 (RFA vs. LTA 1 fibre) $243.65 (RFA vs. LTA 2 fibres)
Utility sources	EQ‐5D‐3L, valued using a utility algorithm based on revealed preferences from the Chinese population.	Mean SF‐36 scores from the literature, mapped to EQ‐5D utility values.	None reported; assumed equal effectiveness.
Total QALYs (Intervention)	0.425	21.31	Assumed equal in all groups
Total QALYs (Comparator)	0.415	21.13	Assumed equal in all groups
Incremental QALYs	0.01	0.18	0
ICER (USD/QALY)^a^	$98 858/QALY	−$14 994/QALY	n.a.
NMB at $50 000 WTP (USD)^a^	−$488.58	$11 699	$2494.54 (RFA vs. lobectomy); $3639.36 (RFA vs. total thyroidectomy); −$714.24 (RFA vs. LTA 1 fibre) −$243.65 (RFA vs. LTA 2 fibres)
NMB at $100 000 WTP (USD)^a^	$11.42	$20 699	$2494.54 (RFA vs. lobectomy); $3639.36 (RFA vs. total thyroidectomy); −$714.24 (RFA vs. LTA 1 fibre) −$243.65 (RFA vs. LTA 2 fibres)
NHB at $50 000 WTP (QALY)^a^	−0.0098	0.234	0.0499 (RFA vs. lobectomy); 0.0728 (RFA vs. total thyroidectomy) −0.01428 (RFA vs. LTA 1 fibre) −0.00487 (RFA vs. LTA 2 fibres)
NHB at $100 000 WTP (QALY)^a^	0.00011	0.207	0.0249 (RFA vs. lobectomy); 0.0364 (RFA vs. total thyroidectomy) −0.00714 (RFA vs. LTA 1 fibre) −0.00244 (RFA vs. LTA 2 fibres)

Abbreviations: ICER, incremental cost‐effectiveness ratio; LTA, laser thermal ablation; n.a., not applicable; NHB, net health benefit; NMB, net monetary benefit; QALY, quality‐adjusted life year; RFA, radiofrequency ablation; USD, United States dollars; WTP, willingness to pay.

^a^ These variables were calculated by the review authors based on available study data.

Yue et al. (2016) conducted a propensity‐score matched cohort study in China, comparing RFA (under local anaesthesia, outpatient) and open total thyroidectomy (inpatient, general anaesthesia) over a 6‐month horizon from a societal perspective (*n* = 108 per group) [25]. The study included both direct and indirect costs, such as productivity loss, and derived utilities using EQ‐5D‐3L data mapped to a Chinese value set. The study provided a detailed cost breakdown sourced from hospital registries and national guidelines, covering medications, surgical materials, and hospital stays. Indirect costs were estimated using GDP per capita as a proxy. Despite these strengths, limitations included the short time horizon, incomplete cost capture (e.g., caregiver time, transport), absence of incremental analysis, and limited sensitivity analysis focused only on RFA unit price, resulting in a moderate methodological quality rating.

Kuo et al. (2023) developed a microsimulation model with a lifetime horizon from a U.S. third‐party perspective, incorporating discounting and probabilistic sensitivity analyses [23]. The model compared RFA (outpatient, local anaesthesia) with lobectomy (inpatient, general anaesthesia) in a base case of a 45‐year‐old woman with a solitary, benign, non‐functioning thyroid nodule. Utility weights were derived by mapping mean SF‐36 scores from literature to EQ‐5D. The study presented broad cost categories without detailed breakdowns and relied on non‐U.S. data sources. Although the model structure and data sources were generally sound, the study was limited by the exclusion of indirect costs (e.g., productivity loss) and limited exploration of structural and methodological uncertainties, resulting in a moderate to high methodological quality rating.

Papi et al. (2023) performed a cost‐minimization analysis using hospital data from Italy, comparing RFA to laser thermal ablation (LTA) and surgery (37 RFA, 120 LTA, 60 surgical, including both hemithyroidectomy and total thyroidectomy) [24]. The analysis adopted a societal perspective and included both direct and some indirect costs based on productivity loss. The study provided a comprehensive cost breakdown from Italian tariffs and hospital records, covering pre‐, intra‐, and post‐operative costs. Although the authors assumed equal clinical effectiveness across interventions, this was not empirically demonstrated, limiting the appropriateness of the cost‐minimization framework. The analysis also used a short time horizon, excluding long‐term follow‐up, and broader societal costs (e.g., caregiver time, transportation), and lacked sensitivity analyses, resulting in a low methodological quality rating.

#### 3.2.2 Economic Outcomes

None of the included studies explicitly reported numeric values for ICERs, NMB, or NHB. However, these values were derived based on the available cost and utility data. Costs and utilities varied considerably across the three studies. The cost‐utility analysis by Yue et al. found that RFA was more costly than surgery with a small QALY gain, resulting in an ICER of $98 858 per QALY, with marginally favourable NMB and NHB estimates at a $100 000 WTP threshold. Kuo et al. showed that RFA was both more effective and less costly, resulting in a negative ICER and favourable NMB and NHB at both $50 000 and $100 000 thresholds. Since Papi et al. assumed equal effectiveness across interventions and did not report QALY estimates, the analysis focused exclusively on costs. Assuming equal effectiveness, NMB and NHB estimates would favour RFA given its lower total costs ($2408.86) compared to hemithyroidectomy ($2494.54) and total thyroidectomy ($3639.36) at both WTP thresholds. LTA was even more cost‐saving, with total costs of $2165.21 when using two fibres and $1694.62 when using a single fibre.

#### 3.2.3 Publication Bias and Certainty Assessment

Publication bias could not be ruled out due to the small number of published studies and the lack of pre‐registration of all three included studies in trial registries such as PROSPERO and ClinicalTrials.gov. The overall certainty of the economic evidence was rated as low. Across the included studies, concerns were identified regarding methodological quality, inconsistency in cost estimates, indirectness due to contextual differences, and imprecision, particularly related to small sample sizes. Full domain‐level assessments are available in Appendix E6.

### 3.3 Secondary Narrative Synthesis

Ten studies provided descriptive cost comparisons between RFA and surgery but did not meet criteria for full economic evaluations. These studies were predominantly retrospective cohort studies (*n* = 7) [27, 28, 30, 31, 32, 34, 35], one prospective cohort study [29], one randomized controlled trial [33] and one time‐driven activity‐based costing model [26]. The ten studies were published between 2014 and 2024 and conducted across various continents: five in Asia (China, Taiwan, Hong Kong) [28, 30, 31, 34, 35], two in North America (United States) [26, 32], one in Europe (Italy) [27], one in South America (Brazil) [33], and one in Africa (Egypt) [29]. Follow‐up durations ranged from 0 to 56 months, with a median of 12 months in studies reporting longitudinal outcomes. A total of 483 RFA cases and 568 thyroid surgery cases were reported across the nine studies with original data, including 246 open lobectomies, 38 open total thyroidectomies, 84 TOETVA lobectomies, and 200 cases in which the surgical subtype was not specified. Sample sizes ranged from 10 to 400 patients per study. RFA was performed as an outpatient procedure in all studies, except for the one modelling study, where the clinical setting was not applicable. Seven of the ten studies reported volume reduction as the primary clinical outcome [27, 28, 29, 30, 31, 34, 35], with a median 12‐month reduction of 78%. Most studies reported low complication rates, with no major adverse events observed in the RFA groups. Further baseline characteristics can be found in Table 2.

**TABLE 2:** Characteristics and outcomes studies included in narrative synthesis.

Study ID	Country	Country income level^a^	Funding source	Study design	Centre	Follow‐up	Sample size RFA	Sample size comparator
Bernardi et al. (2014) [27]	Italy	High‐Income Country	Not reported	Retrospective cohort study	Single centre	12 months	37	74
Che et al. (2015) [28]	China	Upper‐Middle‐Income Country	Not reported	Retrospective cohort study	Not reported	12 months	200	200
Elhefny et al. (2021) [29]	Egypt	Lower‐Middle‐Income	No funding	Prospective cohort study	Single centre	12 months	24	33
Schalch et al. (2021) [33]	Brazil	Upper‐Middle‐Income Country	Not reported	Randomized controlled trial	Single centre	3 months	5	5
Yan et al. (2021) [34]	China	Upper‐Middle‐Income Country	Public funding	Retrospective cohort study	Single centre	Mean follow‐up: 26.2 months	18	35
Ayoub et al. (2023) [26]	United States	High‐Income Country	No funding	Time‐driven activity‐based costing model	n.a.	n.a.	n.a.	n.a.
Miller et al. (2023) [32]	United States	High‐Income Country	Commercial funding	Retrospective cost comparison study	Single centre	0 months, only procedural costs	16	53
Yan et al. (2023) [35]	China	Upper‐Middle‐Income Country	Not reported	Retrospective cohort study	Single centre	Minimum follow‐up of 12 months	49	49
Kuo et al. (2024) [30]	Taiwan	High‐Income Country	Public funding	Retrospective cohort study	Single centre	Mean follow‐up 52.6 months	84	84
Lee et al. (2024) [31]	Hong‐Kong, China	High‐Income Country	Not reported	Retrospective cohort study	Multi‐centre	12 months	50	35

Study	Age, mean (SD or range), years	No. (%) Female sex	Intervention	Comparator	Population characteristics	Details of intervention	Details of comparator
Bernardi et al. (2014) [27]	RFA: 58.3 (3.6); Surgery: 54.9 (3.4)	RFA: 25 (68); Lobectomy: 57 (77)	RFA	Lobectomy	Patients with benign, symptomatic or hyperfunctioning thyroid nodules > 2 cm	Outpatient single‐session RFA, one re‐treatment	Inpatient or short stay lobectomy under general anaesthesia; included pathology analysis
Che et al. (2015) [28]	RFA: 43.8 (12.7) Surgery: 52.4 (13.9)	RFA: 165 (83); Surgery: 154 (77)	RFA	Lobectomy or Thyroidectomy	Adults with benign thyroid nodular goitre (cosmetic issues, symptoms, hyperfunction)	Single‐session RFA; follow‐up at 1, 6, and 12 months	Surgery under general anaesthesia; thyroid lobectomy or total thyroidectomy depending on indication
Elhefny et al. (2021) [29]	RFA: 43.8 (10.2) Surgery: 40.5 (10.9)	RFA: 19 (79); Surgery: 28 (84)	RFA	Thyroidectomy	Adults with symptomatic solitary benign thyroid nodule (TI‐RADS 2–3, Bethesda I–II)	Outpatient RFA under local anaesthesia, 1–2 sessions depending on nodule	Total thyroidectomy under general anaesthesia, inpatient stay
Schalch et al. (2021) [33]	46.4	RFA: 5 (100); Surgery: 5 (100)	RFA	Lobectomy	Patients with benign, predominantly solid (> 50%), symptomatic or aesthetic thyroid nodules > 3 cm, FNAB Bethesda II	Outpatient RFA using local anaesthesia and sedation	Partial thyroidectomy under general anaesthesia
Yan et al. (2021) [34]	RFA: 49.1 (13.8) Surgery: 55.8 (12.9)	RFA: 18 (100); Surgery: 33 (94.3)	RFA	Lobectomy or Thyroidectomy	Patients with benign nodules > 80% solid, developed after prior thyroid surgery; Bethesda II; symptomatic/cosmetic indication	Single outpatient RFA session	General anaesthesia; inpatient; reoperation type varied (completion thyroidectomy, subtotal, lobectomy, or nodule resection)
Ayoub et al. (2023) [26]	n.a.	n.a.	RFA	Lobectomy	Adults with benign non‐functioning thyroid nodules eligible for RFA or lobectomy	RFA in clinic setting, local anaesthesia, with 2 post‐treatment visits	OR lobectomy, general anaesthesia, pre‐op telehealth, 1 post‐op visit
Miller et al. (2023) [32]	Not specified	Not specified	RFA	Lobectomy	Patients with benign or low‐risk thyroid nodules suitable for either surgery or RFA	RFA under general anaesthesia in OR, outpatient setting considered in discussion	Standard lobectomy under general anaesthesia
Yan et al. (2023) [35]	> 60 years, no mean age specified	Not specified	RFA	Lobectomy	Elderly patients with symptomatic benign thyroid nodules	Single‐session RFA in outpatient setting	Lobectomy, with or without isthmusectomy, under general anaesthesia in the operating room.
Kuo et al. (2024) [30]	RFA 47.2 TOETVA 45.6	RFA: 76 (91); TOETVA: 77 (92)	RFA	TOETVA	Patients with unilateral benign thyroid nodules	RFA with high‐res ultrasound guidance, local anaesthesia, outpatient setting	TOETVA with intraoperative nerve monitoring
Lee et al. (2024) [31]	RFA: 52.4 Lobectomy: 53.7	RFA: 41 (82); Lobectomy: 27 (77)	RFA	Lobectomy	Benign, symptomatic thyroid nodules (> 2 cm, confirmed benign cytology); excluded if malignant/toxic/retrosternal	RFA with local anaesthesia, outpatient setting, moving‐shot technique	Open lobectomy under general anaesthesia, inpatient

Study	Incremental costs (2021 US$)	Conclusion costs	Baseline nodule volume RFA, mean (SD or range), mL	Baseline nodule volume comparator, mean (SD or range), mL	Volume reduction in RFA group at 12 months (%)	Complications	RFA retreatment rates, No. (%)
Bernardi et al. (2014) [27]	−$3579.50 (inpatient), −$3063.99 (outpatient)	RFA less costly	12.4 (2.5)	12.8 (2.1)	70	RFA: *n* = 2 (5%)—1 transient voice change, 1 thyroiditis Surgery: transient hypocalcemia (4), nerve palsy (6), wound issues (2), hypothyroidism (23%)	1 (3)
Che et al. (2015) [28]	−$101.43	RFA less costly	5.4 (7.1)	5.9 (6.4)	84.8	RFA: 1% (1 transient hoarseness, 1 rupture). Surgery: *n* = 12 (6%)—2 permanent RLN injuries, 3 transient RLN injuries, 6 transient hypoparathyroidism, 1 hematoma.	Not reported
Elhefny et al. (2021) [29]	$466.31	RFA more costly	12.4 (1.6)	12.0 (2.0)	70	RFA: *n* = 1 (4%)—hypothyroidism; Surgery: *n* = 6 (18%)—2 hoarseness, 1 permanent hypocalcemia, 3 hypothyroidism.	6 (20)
Schalch et al. (2021) [33]	−$2919.67	RFA less costly	Not reported	Not reported	Not reported	RFA: *n* = 3 (60%)—edema, arm paresthesia, hematoma (all self‐limiting); Surgery: *n* = 2 (40%)—1 vocal cord palsy, 1 mild dysphonia.	Not reported
Yan et al. (2021) [34]	−$415.43	RFA less costly	12.8 (17.6)	15.8 (14.8)	85.3	RFA: *n* = 0 (0%)—no complications; Surgery: *n* = 11 (31.4%)—4 transient RLN injuries, 2 persistent RLN injuries, 2 transient hypoparathyroidism, 3 voice changes	0 (0)
Ayoub et al. (2023) [26]	−$10632.92	RFA less costly	Not reported	Not reported	Not reported	Not reported	Not reported
Miller et al. (2023) [32]	−$646.97 to $436.73, depending on probe costs	RFA is cheaper when probe cost is low and performed outpatient	Not reported	Not reported	Not reported	Not reported	Not reported
Yan et al. (2023) [35]	−$260.10	RFA less costly	Median 7.4 (IQR 10.1)	Median 7.1 mL (IQR 13.6)	92.7	RFA: *n* = 0 (0%)—no complications. Surgery: *n* = 13 (26.5%)—10 hypothyroidism, 1 transient RLN palsy, 1 transient hypoparathyroidism, 1 fever.	0 (0)
Kuo et al. (2024) [30]	−$2700.51	RFA less costly	22.8 (20.1)	21.4 (30.1)	77.7	RFA: *n* = 4 (4.8%)—1 hematoma/seroma, 3 tumour ruptures; TOETVA: *n* = 7 (8.3%)—1 infection, 6 subclinical hypothyroidism	Not reported
Lee et al. (2024) [31]	−$5917.27	RFA less costly	15.2 (13.6)	16.6 (20.6)	77	RFA: *n* = 4 (8%)—3 transient neck swelling/bruising, 1 transient vocal cord palsy, no hypothyroidism, no scar issues; Lobectomy: *n* = 13 (37.1%)—2 pitch changes, 2 transient vocal cord palsy, 3 subclinical hypothyroidism, 1 hypothyroidism requiring replacement, 3 wound complications, 2 scar complications	0 (0)

Abbreviations: FNAB, fine needle aspiration biopsy; IQR, interquartile range; LTA, laser thermal ablation; n.a, not applicable; RFA, radiofrequency ablation; RLN, recurrent laryngeal nerve; TI‐RADS, Thyroid Imaging Reporting and Data System; TOETVA, transoral endoscopic thyroidectomy vestibular approach; VRR, volume reduction ratio.

^a^ Country income levels are defined according to the World Bank classification (2024 fiscal year).

Cost estimates varied depending on the healthcare system, procedure type, and inclusion of inpatient versus outpatient settings. Eight of the ten studies concluded that RFA was less costly than surgery [26, 27, 28, 30, 31, 33, 34, 35]. One study found RFA to be more costly [29], while another indicated that this depended on the cost of the RFA probe [32]. After standardizing all values to 2021 U.S. dollars, total RFA costs ranged from $1972 to $8834 (median: $2499), while total surgical costs ranged from $1631 to $19 467 (median: $3579). The overall methodological quality of the cost comparison studies was moderate (Appendix E5), with clear descriptions of populations and costs, but limited adjustment for confounders, description of indirect costs, long‐term follow‐up, and uncertainty analysis.

## 4 Discussion

This systematic review evaluates the economic evidence on RFA for benign, non‐functioning thyroid nodules. Findings suggest that RFA may be associated with favourable cost outcomes compared to surgical management, although estimates vary and certainty remains limited. The findings should be interpreted with caution, given the limited number of full economic evaluations, variation in study quality, and differences in healthcare settings and costing methods. Given these limitations, in the primary synthesis of three studies, two studies show that cost‐effectiveness outcomes ranged from RFA being dominant (more effective and less costly) to an ICER of $98 858 per QALY gained. In the secondary synthesis, 8 out of 10 studies reported lower direct costs for RFA, with cost savings ranging from $100 to over $10 000 per patient depending on setting and comparator. These findings suggest that RFA may be economically favourable compared to surgery, in addition to offering established clinical benefits [36]. However, the available evidence on long‐term effectiveness and utility gains remains limited, which affects confidence in the true economic value of RFA. In addition, short time horizons of most included studies may underestimate long‐term costs of retreatment and surveillance after RFA compared to surgery.

While the study by Kuo et al. applied a rigorous modelling framework, including long‐term projections and probabilistic sensitivity analysis, it was limited by the exclusion of indirect costs and its reliance on utility estimates from non‐U.S. populations [23]. The study demonstrated that RFA was less costly and more effective than lobectomy, resulting in a negative ICER (more effective and less costly) and positive NMB and NHB at conventional willingness‐to‐pay thresholds. This model provides the strongest support available for the cost‐effectiveness of RFA compared to surgery but also illustrates the challenges of extrapolating findings across healthcare systems.

A significant advancement in the broader adoption of RFA for benign thyroid nodules in the United States is the introduction of dedicated Current Procedural Terminology (CPT) codes, effective 1 January 2025 [37]. These codes are expected to streamline reimbursement, improve patient access, and increase clinical uptake, as prior billing limitations had posed a barrier to widespread use. Several major insurers (i.e., Aetna, Cigna, Medicare) have now included RFA in their coverage policies, further supporting its integration into routine care [38, 39, 40]. In earlier economic evaluations, such as the study by Kuo et al., the cost of an RFA procedure was estimated at approximately $5000 [23]. However, with the updated CPT codes and revised reimbursement rates, actual payer costs are now estimated at $2000–$2500. Such a significant reduction may strengthen the cost‐effectiveness profile of RFA over conventional surgical approaches.

In the study by Papi et al., LTA was found to be less costly than RFA, resulting in up to $714 lower total costs per patient [24]. While this suggests that LTA may represent a more economically favourable option, the clinical equivalence between RFA and LTA remains uncertain. The authors assumed equal effectiveness between interventions without reporting utility data, thereby overlooking potential differences in quality of life. Comparative data on quality‐of‐life outcomes between RFA and LTA are lacking, making it difficult to determine whether LTA's lower costs translate into superior cost‐effectiveness.

Future studies should further aim to overcome certain limitations observed in this study. First, one major limitation of this review is the substantial methodological heterogeneity and overall low certainty of the included studies. The economic evaluations varied widely in study design, comparators, analytical perspectives (societal vs. payer), and how outcomes such as costs and QALYs were reported. In addition, there was considerable geographical heterogeneity, as studies were conducted in very different health systems and economies, which limits the direct comparability and generalizability of cost estimates across settings. All three studies did not report key metrics such as ICERs, NMB, or NHB. As a result, reviewers had to estimate these values post hoc using available data and standardized WTP thresholds, which introduces additional uncertainty. Short time horizons, incomplete cost inclusion, and limited sensitivity analyses further reduced the methodological rigour and interpretability of findings. Meta‐analysis was therefore not feasible, and the generalizability of individual study outcomes remains limited. To address this, future research should prioritize economic evaluations that employ a societal perspective, long‐term horizons, and rigorous sensitivity analyses. Additionally, none of the included studies stratified cost‐effectiveness outcomes by patient demographics, comorbidities, or care setting, limiting insights into subgroup‐specific value. Utility values were either indirectly derived from previously published literature (e.g., Kuo et al. [23]) or based on generic instruments such as the EQ‐5D (e.g., Yue et al. [25]). However, EQ‐5D may not adequately capture thyroid‐specific concerns, limiting the precision of QALY‐based estimates [41, 42]. Future studies should incorporate validated, thyroid‐specific preference‐based instruments to improve the accuracy and relevance of utility estimates in this population. A rigorously designed lifetime simulation study that accounts for both direct and indirect costs, including post‐procedural expenses such as long‐term surveillance, potential repeat ablations, and the need for lifelong thyroid hormone replacement after surgery, could provide more robust evidence on the true cost‐effectiveness of RFA for symptomatic benign thyroid nodules.

The recent introduction of CPT codes and expanded insurance coverage in the U.S. are important steps towards broader implementation. However, the real‐world impact of these changes on cost‐effectiveness remains to be fully evaluated. While economic data are now also emerging for indications such as autonomously functioning thyroid nodules [43], data for other potential applications, such as small, low‐risk papillary thyroid cancer, remain scarce. The ongoing expansion of RFA indications underscores the need for robust cost‐effectiveness studies to guide evidence‐based adoption.

## 5 Conclusion

RFA may represent a cost‐effective alternative to surgery for benign thyroid nodules, but high‐quality economic evaluations are lacking, thereby creating an evidence gap that hampers the ability of clinicians and policymakers to fully assess its value. Rigorous, context‐specific cost‐effectiveness studies are needed to determine whether RFA represents a sustainable and value‐based treatment strategy.

## Author Contributions

Conceptualization: Sam P.J. van Dijk and Carrie Cunningham. Methodology: Sam P.J. van Dijk and Carrie Cunningham. Formal analysis: Sam P.J. van Dijk. Investigation: Sam P.J. van Dijk, Matthew M.K. Blanco and Carrie Cunningham. Data curation: Sam P.J. van Dijk and Matthew M.K. Blanco. Writing – original draft: Sam P.J. van Dijk, Matthew M.K. Blanco and Carrie Cunningham. Writing – review and editing: Ali Akhavan, Jessica Liu McMullin, G. Scott Gazelle, Mohammad S. Jalali, Jagpreet Chhatwal, Yinin Hu, van Ginhoven and Rajshri M. Gartland. Supervision: van Ginhoven and Carrie Cunningham. Funding acquisition: Carrie Cunningham.

## Funding

The authors have nothing to report.

## Ethics Statement

The authors have nothing to report.

## Conflicts of Interest

Dr. Cunningham reported receiving personal fees for consulting from Intuitive Robotics and 3M outside the submitted work. No other disclosures were reported.

## Supporting information


**Appendix E1.** Search Strategy.

**Appendix E2.** Data extraction form.

**Appendix E3.** Standardized cost conversion to 2021 USD: Inflation and currency adjustment methods.

**Appendix E4.** Tables of Inflation and Currency Adjustments.

**Appendix E5.** Methodological Quality Assessments.

**Appendix E6.** GRADE Assessment of the Certainty of Economic Evidence.

## Data Availability

Data extracted from included studies and other supporting materials are available from the corresponding author on reasonable request.
