# Supplementary material for: Cost‐Effectiveness of Radiofrequency Ablation for Benign Non‐Functioning Thyroid Nodules: A Systematic Review
Source: Clin Otolaryngol. 2026 Aug 2;51(6):805–18. doi: 10.1111/coa.70150 (PMC13631809; doi:10.1111/coa.70150)
Supplement: Supplementary file 1 [file COA-51-805-s001.pdf]

Appendix E2. Data extraction form

Study information

Study ID	First Author	Year	Country	Country income level (e.g., HIC, UMIC, LMIC)	Funding Source	Journal	Impact factor (2024)

Design & Methods

Study	Study Design	Center	Modelling	Model Type	Model structure	Model assumptions	Model source of parameters	Model Validation	Model Time Horizon	Follow-up	Discount Rate	Perspective

Population & Intervention

Study	Sample Size	Sample size Intervention	Sample size Comparator	Age	Sex	Intervention	Comparator(s)	Population characteristics	Details of intervention	Details of comparator

Utilities

Study	Utility Measure Used	Effectiveness Outcomes	QALYs Reported	Incremental effectiveness (QALYs)

Costs

Currency and Year	Inflation Factor to 2021	2021 Average Exchange Rate	Mean total costs (I, 2021 US$)	Mean total costs (C, 2021 US$)	Incremental costs (2021 US$)

Cost-Effectiveness Analysis Outcomes

Study	Primary outcome	Secondary outcome(s)	ICER Reported	ICER calculated by reviewers	NMB Reported	NMB calculation by reviewers	NHB Reported	NHB calculation by reviewers	WTP Threshold Used	CE Plane or Acceptability Curve	Sensitivity Analysis	Dominance Status

Clinical Outcomes

Study	Baseline nodule volume (I)	Baseline nodule volume (C)	Volume Reduction	Complications	RFA retreatment rates	Procedure duration (I)	Procedure duration (C)	Hospitalization time (I)	Hospitalization time (C)
