# Supplementary material for: Cost‐Effectiveness of Radiofrequency Ablation for Benign Non‐Functioning Thyroid Nodules: A Systematic Review
Source: Clin Otolaryngol. 2026 Aug 2;51(6):805–18. doi: 10.1111/coa.70150 (PMC13631809; doi:10.1111/coa.70150)
Supplement: Supplementary file 2 [file COA-51-805-s002.pdf]

Appendix E3 – Standardized cost conversion to 2021 USD: Inflation and currency adjustment methods

1) Cost standardization: Inflation adjustment in original currencies

To ensure comparability across studies published in different years and reporting costs in various currencies, all cost data were first adjusted for inflation to 2021 values using country-specific indices.

- For studies that reported costs directly in U.S. dollars (USD), we adjusted all values to 2021 USD using the **Consumer Price Index (CPI) for Medical Care** provided by the U.S. Bureau of Labor Statistics (Series ID: *CUUR0000SAM*). Inflation factors were calculated by dividing the 2021 index value (525.276) by the index value of the year in which costs were reported. For example, a study reporting 2018 USD was adjusted using an inflation factor of 525.276 / 484.707 = **1.0837**. Source: https://www.bls.gov/data/ (accessed June 2025).
- For studies that reported costs in euros (EUR), we adjusted all values to 2021 equivalents using the **Harmonized Index of Consumer Prices (HICP)** published by Eurostat. When available, country-specific HICP indices were used to reflect national price developments—e.g., the Italian HICP was used for Bernardi et al. (2014). Inflation correction factors were calculated as the ratio of the HICP index for 2021 to the HICP index of the original cost year. For example, Bernardi et al. reported costs from 2014, and the inflation factor was calculated as 105 / 99.9 = **1.0511**. Source: https://ec.europa.eu/eurostat/databrowser/view/prc_hicp_aind/default/table?lang=en (accessed June 2025).
- For studies reporting costs in Chinese yuan (CNY), we adjusted all values to 2021 equivalents using annual Consumer Price Index (CPI) data from the **National Bureau of Statistics of China**. Inflation correction factors were calculated as the ratio of the 2021 CPI index to the index of the original cost year. For instance, to adjust costs from 2014 to 2021, we used the cumulative product of annual inflation multipliers from 2014 to 2021, resulting in an inflation factor of **1.1646** for Yue et al. Similarly, a factor of **1.1418** was calculated for costs from 2015 as reported in Che et al. Source: https://data.stats.gov.cn/english/easyquery.htm?cn=C01 (accessed June 2025).
- For the study reporting costs in Egyptian pounds (EGP), we adjusted all values to 2021 EGP using annual inflation rates published by Egypt's Central Bank and national statistical authority (CAPMAS). For Elhefny et al. with cost estimates from 2018, we applied a cumulative inflation factor of 1.338909786, based on the year-end inflation rates for 2018 (11.97%), 2019 (7.10%), 2020 (5.43%), and 2021 (5.90%). The cumulative factor was calculated as: 1.1197 × 1.0710 × 1.0543 × 1.0590 = **1.3389**. Inflation rates were retrieved from the Central Bank of Egypt's official website: https://www.cbe.org.eg/en/economic-research/statistics/inflation-rates/historical-data (accessed June 2025).
- To standardize cost estimates reported in Brazilian real (BRL) to 2021 values, we applied national inflation rates using consumer price index (CPI) data. Based on data from Inflation.eu, the annual inflation rate in Brazil was 10.06% in 2021. Accordingly, costs reported in 2020 BRL were multiplied by an inflation factor of **1.1006** to reflect their equivalent 2021 value. The CPI inflation rate was sourced from: Inflation.eu. Historic inflation Brazil – CPI inflation.

https://www.inflation.eu/en/inflation-rates/brazil/historic-inflation/cpi-inflation-brazil.aspx (accessed June 2025).

- For the study reporting costs in Hong Kong dollars (HKD), we adjusted values to 2021 equivalents using the Underlying Composite Consumer Price Index (CPI) published by the **Census and Statistics Department of Hong Kong**. Annual index values for 2019 (100.9), 2020 (102.7), and 2021 (103.3) were retrieved from the official government data portal. For cost estimates from 2019, we applied a cumulative inflation factor of **1.0238**, calculated as: (103.3 / 100.9) = 1.0238. Source: https://www.censtatd.gov.hk/en/web_table.html?id=510-60002A (accessed June 2025).

A full list of annual CPI values we used to calculate the corresponding inflation factors is provided in Supplement Table X.

2) Currency conversion to 2021 U.S. Dollars

After adjusting for inflation in the original currency, all costs were converted to 2021 U.S. dollars (USD). We used the 2021 annual average exchange rates from the World Bank as follows:

Currency	2021 Average Exchange Rate
Chinese Yuan (CNY)	6.45 CNY per USD
Hong Kong Dollar (HKD)	7.77 HKD per USD
Brazilian Real (BRL)	5.39 BRL per USD
Egyptian Pound (EGP)	15.64 EGP per USD
Euro (EUR)	0.85 EUR per USD

These rates represent market exchange values for 2021 and were applied uniformly across all cost estimates converted from local currencies to USD. For studies already reporting in USD, only inflation adjustment was applied using the U.S. Consumer Price Index for Medical Care. Details on inflation correction factors, exchange rates, and final USD-adjusted costs are provided in Supplement Table X.

Source: https://data.worldbank.org/indicator/PA.NUS.FCRF (accessed June 2025).
