# Supplementary material for: Cost‐Effectiveness of Radiofrequency Ablation for Benign Non‐Functioning Thyroid Nodules: A Systematic Review
Source: Clin Otolaryngol. 2026 Aug 2;51(6):805–18. doi: 10.1111/coa.70150 (PMC13631809; doi:10.1111/coa.70150)
Supplement: Supplementary file 3 [file COA-51-805-s003.pdf]

Appendix E6 – GRADE Assessment of the Certainty of Economic Evidence

Domain	Question	Answer
1. **Risk of Bias**	Are there methodological limitations that may reduce confidence in the results (e.g., lack of sensitivity analysis, short time horizon, selection/measurement bias)?	**Yes, moderate to serious concerns.**<br><br>There is considerable variability in methodological quality across the included studies:<br><br>Papi et al. has serious limitations, using a cost-minimization approach based on the unsupported assumption of equal effectiveness, without measuring outcomes or performing sensitivity analyses. Important societal costs were also excluded, leading to a high risk of bias. Yue et al. shows moderate quality, with QALY measurement and a societal perspective. However, the 6-month horizon, limited sensitivity analysis, and no discounting restrict its reliability for long-term evaluation. Kuo et al. is the most robust, using a lifetime microsimulation model, with QALYs, discounting, and multiple sensitivity analyses. Still, it lacks a societal perspective and relies on non-U.S. and literature-based inputs, with incomplete uncertainty analysis.<br><br>Overall, while Kuo et al. demonstrates strong methodological rigor, serious flaws in Papi et al. and moderate limitations in Yue et al. warrant downgrading for risk of bias. The mixed quality across studies indicates a moderate to serious overall risk of bias.
2. **Inconsistency**	Are the results across studies markedly different?	**Yes, some concern due to variability in effect size and cost-effectiveness estimates.**<br><br>Although the three studies point broadly in the same direction, suggesting that radiofrequency ablation (RFA) is economically favorable compared to surgery, there is notable variability in the magnitude and nature of the results that warrants attention.<br><br>In Yue et al., RFA was more costly than surgery with only a marginal gain in QALYs (0.01), resulting in an ICER of $98,858/QALY, which approaches common willingness-to-pay (WTP) thresholds. This suggests borderline cost-effectiveness. In contrast, Kuo et al. found that RFA was both more effective and less costly, leading to a dominant strategy with a negative ICER and strongly favorable net monetary benefit (NMB) and net health benefit (NHB) values. Papi et al., which did not report QALYs, assumed equal effectiveness and demonstrated substantial cost savings for RFA compared to both lobectomy and total thyroidectomy. While useful, this cost-minimization approach omits outcome data, making comparisons more difficult.<br><br>These differences reflect methodological heterogeneity, including divergent time horizons (6 months to lifetime), perspectives (societal vs. third-party payer), and analytic approaches (empirical vs. model-based, cost-utility vs. cost-minimization). Moreover, total costs vary widely across studies (e.g., $2,400 in Papi vs. $16,500 in Kuo), and the ICERs range from -$14,994 to nearly $100,000 per QALY, indicating substantial inconsistency in the estimated value-for-money.

3. **Indirectness**	Is the population, intervention, comparator, or outcome not directly applicable to your research question?	**Yes, some concerns.**<br><br>All three studies focus on patients with benign thyroid nodules, aligning with the target population. However, indirectness arises from variation in setting, comparators, and outcome measurement.<br><br>The studies were conducted in China, the U.S., and Italy, each with different healthcare systems and cost structures. Comparators varied, open thyroidectomy (Yue), lobectomy (Kuo), and both lobectomy and total thyroidectomy (Papi), which may affect outcomes and costs. Furthermore, only two studies measured QALYs, while Papi et al. assumed equal effectiveness, limiting comparability.<br><br>While the clinical question is broadly addressed, these differences introduce some concerns regarding applicability.
4. **Imprecision**	Are the estimates imprecise (e.g., wide confidence intervals, small sample sizes, minimal QALY gains)?	**Yes, some concerns.**<br><br>Sample sizes were relatively small (e.g., n = 108 per group in Yue et al.; n = 37 RFA patients in Papi et al.), limiting statistical power.<br>Moreover, the incremental QALY gains were minimal (0.01 in Yue et al. and 0.18 in Kuo et al.) raising questions about the clinical relevance and robustness of the effect size.
5. **Publication Bias**	Is there a risk that relevant studies have not been published?	**Unclear, possible concern.**<br><br>Economic evaluations are rarely registered, and the three included studies were not pre-registered. A search in PROSPERO yielded no relevant systematic reviews, and although two trials evaluating the cost of RFA versus surgery were registered recently (2023 and 2025), they are likely still ongoing. No unpublished conference abstracts or grey literature were identified during the search, and no prior systematic reviews on this topic were found.<br><br>While no direct evidence of publication bias was observed, the limited number of published studies, all showing favorable or cost-saving results for RFA, could be a potential concern for selective publication.
