# Supplementary material for: Cost‐Effectiveness of Radiofrequency Ablation for Benign Non‐Functioning Thyroid Nodules: A Systematic Review
Source: Clin Otolaryngol. 2026 Aug 2;51(6):805–18. doi: 10.1111/coa.70150 (PMC13631809; doi:10.1111/coa.70150)
Supplement: Supplementary file 4 [file COA-51-805-s004.pdf]

Appendix E5 – Methodological Quality Assessments

CHEC Item	Yue et al.	Papi et al.	Notes
1. Study population clearly described?	Yes	Yes	Yue et al. less detailed
2. Competing alternatives clearly described?	Yes	Yes	Both clearly described
3. Research question posed?	Yes	Yes	Both clearly posed
4. Appropriate economic study design?	Yes	No	Papi et al.: CMA with unproven equal effectiveness assumption
5. Appropriate time horizon?	No	No	Both too short
6. Appropriate perspective?	Yes	Yes	Both societal but limited
7. All relevant costs identified?	No	No	Both missing some societal costs
8. Costs measured appropriately?	Yes	Yes	Both clearly measured
9. Costs valued appropriately?	Yes	Yes	Both with clear sources
10. Relevant outcomes identified?	Yes	No	Papi et al.: no effectiveness outcomes, only costs
11. Outcomes measured appropriately?	Yes	No	Papi et al.: outcomes assumed
12. Outcomes valued appropriately?	Yes	No	Papi et al.: assumed, no data
13. Incremental analysis performed?	No	No	Neither performed incremental analysis
14. Discounting applied?	No	No	Neither applied discounting
15. Sensitivity analysis conducted?	No	No	Neither conducted sensitivity analyses
16. Conclusions supported by data?	Yes	Yes	Both justified
17. Generalizability discussed?	Yes	No	Papi et al. lacks
18. Conflict of interest stated?	Yes	Yes	Both stated
19. Ethical/distributional issues discussed?	No	No	Neither adressed

Final assessment per study

Yue et al. (2016):

- Strengths: Clear comparison, costs and QALYs measured, societal perspective.
- Weaknesses: Short horizon, incomplete sensitivity analysis, missing broader cost components.
- Overall: Moderate methodological quality

Papi et al. (2023):

- Strengths: Detailed cost breakdown, transparent methods.
- Weaknesses: Cost-minimization analysis based on flawed assumption of equal effectiveness, no outcome measurement, no sensitivity analysis, narrow cost perspective.
- Overall: Low methodological quality

Overall rating for the combined evidence:
Moderate methodological quality, with notable variation in study rigor.

CHEC Criteria List – Yue et al.

	Item	Yes	No	Extract
1.	Is the study population clearly described?	X		The study population is clearly described (inclusion criteria: benign nodules, exclusion: malignancy), though no detailed information on drop-outs.
2.	Are competing alternatives clearly described?	X		RFA and open thyroidectomy (OT) are clearlydescribed, including their context and indication.
3.	Is a well-defined research question posed in answerable form?	X		Comparison of HRQoL and cost-effectiveness of RFA vs. OT for BTNs.
4.	Is the economic study design appropriate to the stated objective?	X		Cost-effectiveness analysis from a societal perspective.
5.	Is the chosen time horizon appropriate to include relevant costs and consequences?		X	The time horizon of 6 months is too short to capture long-term RFA complications or re-treatment.
6.	Is the actual perspective chosen appropriate?	X		The study adopts a societal perspective (including both direct and indirect costs), which is appropriate.
7.	Are all important and relevant costs for each alternative identified?		X	Direct and indirect costs described; however transport costs, caregiver time and costs are not included.
8.	Are all costs measured appropriatelyin physical units?	X		Costs in physical units, with rates from hospitalizations, materials, etc.
9.	Are costs valued appropriately?	X		Costs presented in CNY and USD with exchange rate, from public sources.
10.	Are all important and relevant outcomes for each alternative identified?	X		HRQoL (SF-36 and EQ-5D-3L) and QALYs measured.
11.	Are all outcomes measured appropriately?	X		Validated SF-36, EQ-5D-3L, trapezoidal method QALYs.
12.	Are outcomes valued appropriately?	X		EQ-5D-3L utility scores based on Chinese value set.
13.	Is an incremental analysis of costs and outcomes of alternatives performed?		X	No explicit incremental analysis provided; only total costs and QALYs per arm are reported. Reviewers had to calculate the ICER and other incremental measures.
14.	Are all future costs and outcomes discounted appropriately?		X	No discounting applied (6-month horizon).
15.	Are all important variables, whose values are uncertain, appropriately subjected to sensitivity analysis?		X	The authors conducted sensitivity analysis on the unit price of RFA but did not examine variability in key parameters such as willingness-to-pay thresholds, discount rates, or clinical outcomes (including complication rates and retreatment probabilities).
16.	Do the conclusions follow from the data reported?	X		The conclusions are consistent with the presented data, though with limitations acknowledged.
17.	Does the study discuss the generalizability of the results to other settings and patient/ client groups?	X		The study explicitly discusses the applicabilityto other settings, especially outside China.
18.	Does the article indicate that there is no potential conflict of interest of study researcher(s) and funder(s)?	X		Statement of “no conflicts of interest.”
19.	Are ethical and distributional issues discussed appropriately?		X	There is no discussion of ethical or distributional issues (e.g., disparities in access or socioeconomic factors).

CHEC Criteria List – Papi et al.

	Item	Yes	No	Extract
1.	Is the study population clearly described?	X		Inclusion criteria, age, sex, number of procedures, follow-up provided.
2.	Are competing alternatives clearly described?	X		MITT (RFA/LTA) versus traditional surgeryexplicitly described, including costs and procedures.
3.	Is a well-defined research question posed in answerable form?	X		Clear aim to compare costs of MITT versus surgery.
4.	Is the economic study design appropriate to the stated objective?		X	The study uses a cost-minimization analysis based on an assumption of equal effectiveness between MITT and surgery. However, evidence suggests that quality of life differences exist between these interventions. The assumption of equivalence oversimplifies the comparison and limits the appropriateness of the economic design.
5.	Is the chosen time horizon appropriate to include relevant costs and consequences?	X		12 month time horizon. No long-term follow-up or regrowth costs.
6.	Is the actual perspective chosen appropriate?	X		Perspective societal, direct and limited indirect costs considered. The study did not consider broader societal factors such as caregiver time, transportation costs, or patient-incurred costs.
7.	Are all important and relevant costs for each alternative identified?		X	Hospitalization, procedure, personnel, and indirect costs included, but broader societal costs (e.g., out-of-pocket, productivity losses of caregivers) not fully captured.
8.	Are all costs measured appropriatelyin physical units?	X		Costs in euros with detailed components (instruments, drugs, hospital stay).
9.	Are costs valued appropriately?	X		Based on hospital registries, national guidelines, and specific cost sources.
10.	Are all important and relevant outcomes for each alternative identified?		X	Did not consider QoL differences or clinical effectiveness (assumed equal effectiveness).
11.	Are all outcomes measured appropriately?		X	Outcomes were not measured but assumed; cost-minimization analysis used.
12.	Are outcomes valued appropriately?		X	No valuation; assumes equal effectiveness.
13.	Is an incremental analysis of costs and outcomes of alternatives performed?		X	No formal incremental analysis (costs vs. outcomes), only cost comparison.
14.	Are all future costs and outcomes discounted appropriately?		X	Discounting not applicable or reported due to short time horizon.
15.	Are all important variables, whose values are uncertain, appropriately subjected to sensitivity analysis?		X	No sensitivity analysis; direct cost differences reported, but no variation analysis for uncertain variables.
16.	Do the conclusions follow from the data reported?	X		Cost-minimization conclusions justified by data.
17.	Does the study discuss the generalizability of the results to other settings and patient/ client groups?		X	Focused on a single center in Italy, no detailed discussion on generalizabilityto other contexts.
18.	Does the article indicate that there is no potential conflict of interest of study researcher(s) and funder(s)?	X		Authors declared no competing interests.
19.	Are ethical and distributional issues discussed appropriately?		X	Ethical aspects and potential distributional concerns (e.g., equity) not addressed.

Kuo et al. Strengths:

- Robust microsimulation model aligned with disease pathways.
- Lifetime horizon used, with QALYs and appropriate 3% discounting.
- Included deterministic and probabilistic sensitivity analyses.

Weaknesses:

- Narrow third-party payer perspective without justification; societal costs not included.
- Incomplete uncertainty analysis — structural and methodological uncertainties not fully explored.
- Relied on literature-based inputs; lacked real-world or patient-level data.
- Some input data (e.g., utilities, transition probabilities) were based on non-U.S. or limited-quality sources.

Overall:

A high-quality model-based evaluation with strong technical features, but limited by a narrow perspective, incomplete uncertainty exploration, and data limitations.

The ECOBIAS checklist for bias in economic evaluation.

Kuo et al.

	Type of bias	Issues addressed *(questions to consider)*	Relevant to study Yes/No/Partly/Unclear/NA	How did you deal with this bias? *(description of strategy and rationale)*
**PART A. Overall checklist for bias in economic evaluation**				
	Narrow perspective bias	Was a societal perspective adopted? If not, has a different perspective been justified?	**No**. Kuo et al. used a third-party payer perspective, not a societal perspective, and did not justify this choice.	Ideally, the societal perspective would have included indirect costs. This limitation is highlighted in the synthesis, and it is recommended that future studies adopt a societal perspective for a more comprehensive evaluation.
	Inefficient comparator bias*	Was the best alternative chosen as comparator? Was current practice chosen as a comparator? Have all comparators been described in sufficient detail?	**Yes.** The comparator was standard surgical care (lobectomy), which is the typical current practice. Descriptions were adequate.	
	Cost measurement omission bias	Were all costs relevant to the disease and intervention identified and considered?	**Partly**. Only direct costs were included; indirect costs such as productivity loss were excluded.	We highlight this limitation and recommend future models to include these costs for completeness.
	Intermittent data collection bias	Was the resource use measured continuously?	**No**. The study used a model based on literature estimates rather than empirical, continuously measured resource use data. This may limit accuracy and applicability of resource and cost estimates.	We acknowledge this limitation and suggest future studies use patient-level data to capture real-world resource use more precisely, if available.
	Invalid valuation bias	Is the price calculation presented in a detailed manner? Have reference prices been used?	**Yes**. Prices were presented in detail, converted to 2021 USD using the Consumer Price Index.	
	Ordinal ICER bias	Have cardinal scales for the outcomes measure in a CEA been used?	**Yes.** Outcomes were measured in QALYs, which use cardinal scales.	

The ECOBIAS checklist for bias in economic evaluation.

Kuo et al.

	Type of bias	Issues addressed *(questions to consider)*	Relevant to study Yes/No/Partly/Unclear/NA	How did you deal with this bias? *(description of strategy and rationale)*
	Double-counting bias	Are variables adequately checked for double-counting?	**Unclear.** Kuo et al. did not explicitly describe measures to prevent double-counting of costs or effects in their microsimulation model.	We recommend interpreting the cost-effectiveness results with caution, recognizing the possibility of overestimation if certain resources or effects were inadvertently counted multiple times.
	Inappropriate discounting bias	Have discounting rates from guidelines been applied?	**Yes.** Discounting was applied at a 3% annual rate.	
	Limited sensitivity analysis bias§	Have the four principles of uncertainty (methodological, structural, heterogeneity, parameter) been considered in sufficient detail?	**No.** Both deterministic and probabilistic sensitivity analyses were performed, but structural uncertainty(e.g., different model structures) and methodological uncertainty (e.g., choice of time horizon or discount rates) were not fully explored or reported.	This limitation is acknowledged, and future analyses should explore alternative model structures and parameters to capture all relevant uncertainties.
	Sponsor bias	Have sponsorships been disclosed? Is the study protocol freely accessible?	**Yes.** The research did not receive any specific grant from funding agencies in the public, commercial, or nonprofit sectors.	
	Reporting and dissemination bias	Has the study/trial been listed in a trial register? Have all results been reported according to the study protocol?	**Unsure,** there is no mention of trial registration.	
**PART B. Model-specific aspects of bias in economic evaluation**				
I Bias related to structure				

The ECOBIAS checklist for bias in economic evaluation.

Kuo et al.

	Type of bias	Issues addressed<br>*(questions to consider)*	Relevant to study<br>Yes/No/Partly/Unclear/NA	How did you deal with this bias?<br>*(description of strategy and rationale)*
	Structural assumptions bias	Is the model structure in line with coherent theory? Do treatment pathways reflect the nature of disease?	**Yes.** Model structure (microsimulation) aligned with disease pathways, assumptions clearly stated.	
	No treatment comparator bias*	Is there an adequate comparator, i.e. care as usual?	**Yes.** The comparator was standard care; reasonable.	
	Wrong model bias	Is the model chosen adequate regarding the decision problem?	**Yes.** Microsimulation is appropriate for this decision problem.	
	Limited time horizon bias	Was a lifetime horizon chosen? Were shorter time horizons adequately justified?	**Yes.** Lifetime horizon was used, appropriate for this evaluation.	
II Bias related to data				
	Bias related to data identification	Are the methods of data identification transparent? Are all choices justified adequately? Do the input parameters come from high-quality and well-designed studies?	**Partly.** Sources for transition probabilities and utilities were reported, but some were based on limited or non-U.S. data.	
	Bias related to baseline data	Are probabilities, for example, based on natural history data? Is transformation of rates into transition probabilities done accurately?	**Partly.** Transition probabilities derived from literature, but extrapolation or adjustments were not always explicit.	
	Bias related to treatment effects	Are relative treatment effects synthesized using appropriate meta-analytic techniques? Are extrapolations documented and well justified? Are alternative assumptions explored regarding extrapolation?	**Partly.** No meta-analyses were used, and the assumptions for extrapolation were not thoroughly justified.	
	Bias related to quality-of-life weights (utilities)	Are the utilities incorporated appropriate for the specific decision problem?	**Partly.** Utilities were derived from mappings and assumptions due to lack of direct U.S. data.	

The ECOBIAS checklist for bias in economic evaluation.

Kuo et al.

	Type of bias	Issues addressed *(questions to consider)*	Relevant to study Yes/No/Partly/Unclear/NA	How did you deal with this bias? *(description of strategy and rationale)*
	Non-transparent data incorporation bias	Is the process of data incorporation transparent? Are all data and their sources described in detail?	**Yes.** Most data sources were detailed.	
	Limited scope bias§	Have the four principles of uncertainty (methodological, structural, heterogeneity, parameter) been considered?	**Yes.** Four uncertainty principles were considered, but not comprehensively detailed.	
III Bias related to consistency				
	Bias related to internal consistency	Has internal consistency in terms of mathematical logic been evaluated?	**Yes**. The model's mathematical logic and calculations appeared sound.	

*These biases are overlapping regarding their content.

§These biases are overlapping regarding their content.

Newcastle-Ottawa Scale (NOS) Quality Assessment Summary for Included Cohort Studies

Study	Selection (4)	Comparability (2)	Outcome (3)	Overall (9)	Interpretation
Bernardi et al. (2014)	4	0	3	7	Moderate to high quality (some risk of bias)
Che et al. (2015)	4	0	3	7	Moderate to high quality (some risk of bias)
Elhefny et al. (2021)	4	0	3	7	Moderate to high quality (some risk of bias)
Yan et al. (2021)	4	0	3	7	Moderate to high quality (some risk of bias)
Yan et al. (2023)	4	2	3	9	High quality (low risk of bias)
Kuo et al. (2024)	4	2	3	9	High quality (low risk of bias)
Lee et al. (2024)	4	0	3	7	Moderate to high quality (some risk of bias)

Overall rating for combined evidence

- Moderate to high methodological quality, with considerable variation in study rigor and especially in the gathering of cost data.
- Most studies achieved high selection and outcome domain scores but lacked adjustment for confounding (comparability). While cost data were often well-documented in hospital records, broader societal costs and long-term outcomes were frequently underreported or excluded.

NEWCASTLE - OTTAWA QUALITY ASSESSMENT SCALE
COHORT STUDIES

Bernardi et al.

Selection

1) Representativeness of the exposed cohort
   a) ~~truly representative of the average __________________(describe) in the community~~
   b) somewhat representative of the average ________________inthe community ❈
   c) ~~selected group of users eg nurses, volunteers~~
   d) ~~no description of the derivation of the cohort~~

2) Selection of the non exposed cohort
   a) drawn from the same community as the exposed cohort ❈
   b) ~~drawn from a different source~~
   c) ~~no description of the derivation of the non exposed cohort~~

3) Ascertainment of exposure
   a) secure record (eg surgical records) ❈
   b) ~~structured interview~~
   c) ~~written self report~~
   d) ~~no description~~

4) Demonstration that outcome of interest was not present at start of study
   a) yes ❈
   b) ~~no~~

Comparability

1) Comparability of cohorts on the basis of the design or analysis
   a) ~~study controls for key baseline factors (e.g., nodule size, function, symptoms).~~
   b) ~~study controls for any additional factor (This criteria could be modified to indicate specific control for a second important factor.)~~

Outcome

1) Assessment of outcome
   a) ~~independent blind assessment~~
   b) record linkage ❈
   c) ~~self report~~
   d) ~~no description~~

2) Was follow-up long enough for outcomes to occur
   a) yes (12 months) ❈
   b) ~~no~~

3) Adequacy of follow up of cohorts
   a) complete follow up - all subjects accounted for ❈
   b) ~~subjects lost to follow up unlikely to introduce bias - small number lost - > ________ % (select an adequate %) follow up, or description provided of those lost)~~
   c) ~~follow up rate < ______ % (select an adequate %) and no description of those lost~~
   d) ~~no statement~~

NEWCASTLE - OTTAWA QUALITY ASSESSMENT SCALE
COHORT STUDIES

Che et al.

Selection

1) Representativeness of the exposed cohort
   a) ~~truly representative of the average ________________ (describe) in the community~~
   b) somewhat representative of the average patient with thyroid nodules in the community ❋
   c) ~~selected group of users eg nurses, volunteers~~
   d) ~~no description of the derivation of the cohort~~

2) Selection of the non exposed cohort
   a) drawn from the same community as the exposed cohort ❋
   b) ~~drawn from a different source~~
   c) ~~no description of the derivation of the non exposed cohort~~

3) Ascertainment of exposure
   a) secure record (eg surgical records) ❋
   b) ~~structured interview~~
   c) ~~written self report~~
   d) ~~no description~~

4) Demonstration that outcome of interest was not present at start of study
   a) yes ❋
   b) ~~no~~

Comparability

1) Comparability of cohorts on the basis of the design or analysis
   a) ~~study controls for ______________ (select the most important factor)~~
   b) ~~study controls for any additional factor (This criteria could be modified to indicate specific control for a second important factor.)~~

Outcome

1) Assessment of outcome
   a) ~~independent blind assessment~~
   b) record linkage ❋
   c) ~~self report~~
   d) ~~no description~~

2) Was follow-up long enough for outcomes to occur
   a) yes (select an adequate follow up period for outcome of interest) ❋
   b) ~~no~~

3) Adequacy of follow up of cohorts
   a) complete follow up - all subjects accounted for ❋
   b) ~~subjects lost to follow up unlikely to introduce bias - small number lost - > _______ % (select an adequate %) follow up, or description provided of those lost)~~
   c) ~~follow up rate < _____ % (select an adequate %) and no description of those lost~~
   d) ~~no statement~~

NEWCASTLE - OTTAWA QUALITY ASSESSMENT SCALE
COHORT STUDIES

Elhefny et al.

Selection

1) Representativeness of the exposed cohort
   a) ~~truly representative of the average ________________(describe) in the community~~
   b) somewhat representative of the average _______________ in the community ❈
   c) ~~selected group of users eg nurses, volunteers~~
   d) ~~no description of the derivation of the cohort~~

2) Selection of the non exposed cohort
   a) drawn from the same community as the exposed cohort ❈
   b) ~~drawn from a different source~~
   c) ~~no description of the derivation of the non exposed cohort~~

3) Ascertainment of exposure
   a) secure record (eg surgical records) ❈
   b) ~~structured interview~~
   c) ~~written self report~~
   d) ~~no description~~

4) Demonstration that outcome of interest was not present at start of study
   a) yes ❈
   b) ~~no~~

Comparability

1) Comparability of cohorts on the basis of the design or analysis
   a) ~~study controls for _______________(select the most important factor)~~
   b) ~~study controls for any additional factor (This criteria could be modified to indicate specific control for a second important factor.)~~

Outcome

1) Assessment of outcome
   a) ~~independent blind assessment~~
   b) record linkage ❈
   c) ~~self report~~
   d) ~~no description~~

2) Was follow-up long enough for outcomes to occur
   a) yes (select an adequate follow up period for outcome of interest) ❈
   b) ~~no~~

3) Adequacy of follow up of cohorts
   a) complete follow up - all subjects accounted for ❈
   b) ~~subjects lost to follow up unlikely to introduce bias - small number lost - > ________% (select an adequate %) follow up, or description provided of those lost)~~
   c) ~~follow up rate < ______% (select an adequate %) and no description of those lost~~
   d) ~~no statement~~

NEWCASTLE - OTTAWA QUALITY ASSESSMENT SCALE
COHORT STUDIES

Kuo et al. 2024

Selection

1) Representativeness of the exposed cohort
   a) ~~truly representative of the average ________________(describe) in the community~~
   b) somewhat representative of the average _______________in the community ❈
   c) ~~selected group of users eg nurses, volunteers~~
   d) ~~no description of the derivation of the cohort~~

2) Selection of the non exposed cohort
   a) drawn from the same community as the exposed cohort ❈
   b) ~~drawn from a different source~~
   c) ~~no description of the derivation of the non exposed cohort~~

3) Ascertainment of exposure
   a) secure record (eg surgical records) ❈
   b) ~~structured interview~~
   c) ~~written self report~~
   d) ~~no description~~

4) Demonstration that outcome of interest was not present at start of study
   a) yes ❈
   b) ~~no~~

Comparability

1) Comparability of cohorts on the basis of the design or analysis
   a) study controls for Yes, via propensity score matching on age, sex, and nodule size. ❈
   b) study controls for any additional factors: **Yes, via propensity score matching on age, sex, and nodule size.** ❈

Outcome

1) Assessment of outcome
   a) ~~independent blind assessment~~
   b) record linkage ❈
   c) ~~self report~~
   d) ~~no description~~

2) Was follow-up long enough for outcomes to occur
   a) yes (select an adequate follow up period for outcome of interest) ❈
   b) ~~no~~

3) Adequacy of follow up of cohorts
   a) complete follow up - all subjects accounted for ❈
   b) ~~subjects lost to follow up unlikely to introduce bias - small number lost - > _______% (select an adequate %) follow up, or description provided of those lost)~~
   c) ~~follow up rate < _____% (select an adequate %) and no description of those lost~~
   d) ~~no statement~~

NEWCASTLE - OTTAWA QUALITY ASSESSMENT SCALE
COHORT STUDIES

Lee et al.

Selection

1) Representativeness of the exposed cohort
   a) ~~truly representative of the average ________________(describe) in the community~~
   b) somewhat representative of the average ______________inthe community ❈
   c) ~~selected group of users eg nurses, volunteers~~
   d) ~~no description of the derivation of the cohort~~

2) Selection of the non exposed cohort
   a) drawn from the same community as the exposed cohort ❈
   b) ~~drawn from a different source~~
   c) ~~no description of the derivation of the non exposed cohort~~

3) Ascertainment of exposure
   a) secure record (eg surgical records) ❈
   b) ~~structured interview~~
   c) ~~written self report~~
   d) ~~no description~~

4) Demonstration that outcome of interest was not present at start of study
   a) yes ❈
   b) ~~no~~

Comparability

1) Comparability of cohorts on the basis of the design or analysis
   a) ~~study controls for ______________(select the most important factor)~~
   b) ~~study controls for any additional factor (This criteria could be modified to indicate specific control for a second important factor.)~~

Outcome

1) Assessment of outcome
   a) ~~independent blind assessment~~
   b) record linkage ❈
   c) ~~self report~~
   d) ~~no description~~

2) Was follow-up long enough for outcomes to occur
   a) yes (select an adequate follow up period for outcome of interest) ❈
   b) ~~no~~

3) Adequacy of follow up of cohorts
   a) complete follow up - all subjects accounted for ❈
   b) ~~subjects lost to follow up unlikely to introduce bias - small number lost - > _______% (select an adequate %) follow up, or description provided of those lost)~~
   c) ~~follow up rate < _____% (select an adequate %) and no description of those lost~~
   d) ~~no statement~~

NEWCASTLE - OTTAWA QUALITY ASSESSMENT SCALE
COHORT STUDIES

Yan et al. 2021

Selection

1) Representativeness of the exposed cohort
   a) ~~truly representative of the average ________________(describe) in the community~~
   b) somewhat representative of the average _______________ in the community ❋
   c) ~~selected group of users eg nurses, volunteers~~
   d) ~~no description of the derivation of the cohort~~

2) Selection of the non exposed cohort
   a) drawn from the same community as the exposed cohort ❋
   b) ~~drawn from a different source~~
   c) ~~no description of the derivation of the non exposed cohort~~

3) Ascertainment of exposure
   a) secure record (eg surgical records) ❋
   b) ~~structured interview~~
   c) ~~written self report~~
   d) ~~no description~~

4) Demonstration that outcome of interest was not present at start of study
   a) yes ❋
   b) ~~no~~

Comparability

1) Comparability of cohorts on the basis of the design or analysis
   a) ~~study controls for _______________(select the most important factor)~~
   b) ~~study controls for any additional factor (This criteria could be modified to indicate specific control for a second important factor.)~~

Outcome

1) Assessment of outcome
   a) ~~independent blind assessment~~
   b) record linkage ❋
   c) ~~self report~~
   d) ~~no description~~

2) Was follow-up long enough for outcomes to occur
   a) yes (select an adequate follow up period for outcome of interest) ❋
   b) ~~no~~

3) Adequacy of follow up of cohorts
   a) complete follow up - all subjects accounted for ❋
   b) ~~subjects lost to follow up unlikely to introduce bias - small number lost - > ________% (select an adequate %) follow up, or description provided of those lost)~~
   c) ~~follow up rate < ______% (select an adequate %) and no description of those lost~~
   d) ~~no statement~~

NEWCASTLE - OTTAWA QUALITY ASSESSMENT SCALE
COHORT STUDIES

Yan et al. 2023

Selection

1) Representativeness of the exposed cohort
   a) ~~truly representative of the average ________________(describe) in the community~~
   b) somewhat representative of the average ______________ in the community ❋
   c) ~~selected group of users eg nurses, volunteers~~
   d) ~~no description of the derivation of the cohort~~

2) Selection of the non exposed cohort
   a) drawn from the same community as the exposed cohort ❋
   b) ~~drawn from a different source~~
   c) ~~no description of the derivation of the non exposed cohort~~

3) Ascertainment of exposure
   a) secure record (eg surgical records) ❋
   b) ~~structured interview~~
   c) ~~written self report~~
   d) ~~no description~~

4) Demonstration that outcome of interest was not present at start of study
   a) yes ❋
   b) ~~no~~

Comparability

1) Comparability of cohorts on the basis of the design or analysis
   a) study controls for: they used propensity score matching (PSM), adjusting for key confounders (age, sex, nodule size, volume, location, TI-RADS score). ❋
   b) study controls for any additional factor ❋ **they used propensity score matching (PSM), adjusting for key confounders (age, sex, nodule size, volume, location, TI-RADS score).**

Outcome

1) Assessment of outcome
   a) ~~independent blind assessment~~
   b) record linkage ❋
   c) ~~self report~~
   d) ~~no description~~

2) Was follow-up long enough for outcomes to occur
   a) yes (select an adequate follow up period for outcome of interest) ❋
   b) ~~no~~

3) Adequacy of follow up of cohorts
   a) complete follow up - all subjects accounted for ❋
   b) ~~subjects lost to follow up unlikely to introduce bias - small number lost - > ________ % (select an adequate %) follow up, or description provided of those lost)~~
   c) ~~follow up rate < ______ % (select an adequate %) and no description of those lost~~
   d) ~~no statement~~

CHEERS 2022 Narrative appraisal – Schalch et al. (2021)

Study type: Randomized cost comparison study
Population: 10 patients (5 RFA, 5 lobectomy)
Country: Brazil
No health outcomes or utilities reported → not a full economic evaluation

Key CHEERS items well reported:

- Title and objectives are clearly described.
- Study population, setting, and interventions are well documented.
- Costing methods are appropriately described using Brazilian hospital systems (SIMPRO, CBHPM).
- Currency and price year are clearly reported (Brazilian Real, 2020).
- Results are clearly summarized in tables.

Partially reported or missing:

- Perspective is not explicitly stated.
- Time horizon is very short (in-hospital only) and not justified.
- Uncertainty is minimally addressed; only probe cost variation is explored.
- No sensitivity analyses, no economic analysis plan, and no discussion of equity or heterogeneity.
- No health outcomes, utilities, or cost-effectiveness metrics (e.g., ICERs) are presented.

Summary statement:

Schalch et al. (2021) reports a small, randomized cost comparison of RFA and lobectomy with clear documentation of population, setting, and cost data, but lacks outcomes, sensitivity analysis, and a defined perspective—therefore not qualifying as a full economic evaluation.

CHEERS 2022 Narrative Appraisal – Miller et al. (2022)
Study type: Retrospective cost comparison
Population: 53 open lobectomy vs. 16 RFA procedures
Country: United States
No health outcomes or utility data reported → not a full economic evaluation

Key CHEERS items well reported:

- Title and objectives are clearly described.
- Patient characteristics, inclusion criteria, and institutional context described.
- Variable direct costs defined and transparently sourced from institutional systems.
- Costs in U.S. dollars; implicit date range of 2016–2020.
- Open lobectomy vs. RFA clearly outlined.
- Costs are clearly presented and include sensitivity to probe pricing.

Partially reported or missing:

- Perspective is not explicitly stated.
- No follow-up, time horizon not justified.
- Uncertainty: Only probe cost variation modeled; no sensitivity or scenario analyses beyond this.
- Heterogeneity & equity: not addressed.
- Outcomes: No clinical effectiveness, utilities, or ICERs reported.
- Engagement: No mention of patient or stakeholder involvement.
- Analysis Plan & Discounting: No economic analysis plan reported; discounting not applicable due to short time frame.

Summary statement:

Miller et al. (2022) provides a clear retrospective cost comparison of RFA and lobectomy using institutional VDC data. However, the absence of health outcomes, defined perspective, time horizon justification, and broader uncertainty analysis limits its classification to a partial economic evaluation.

CHEERS 2022 Narrative Appraisal – Ayoub et al. (2023)
Study type: Retrospective cost analysis
Population: 129 patients (65 RFA, 64 surgery)
Country: United States
No health outcomes or utilities reported → not a full economic evaluation

Key CHEERS items well reported:

• Title and objectives are clearly described.
• Study population, setting, and interventions are well documented.
• Costing methods are transparently described using institutional billing data.
• Currency and price year are reported (USD, based on 2021 billing).
• Results are clearly presented with statistical comparisons between groups**.**

Partially reported or missing:

• Perspective is not explicitly stated.
• Time horizon is limited to procedural episode; no justification is provided.
• Uncertainty is partially addressed through subgroup and multivariate analyses, but no formal sensitivity analyses are included.
• No discussion of equity or heterogeneity beyond age/sex.
• No health outcomes, utilities, or cost-effectiveness metrics (e.g., ICERs) are presented.

Summary statement:

Ayoub et al. (2023) conducted a retrospective cost analysis using time-driven activity-based costing (TDABC) to compare RFA and surgery for benign thyroid nodules. The study offers detailed, methodologically sound costing grounded in clinical workflows, but lacks outcome measures, an explicit perspective, time horizon justification, and formal sensitivity analysis—therefore not qualifying as a full economic evaluation.
