# Supplementary material for: Cost‐Effectiveness of Radiofrequency Ablation for Benign Non‐Functioning Thyroid Nodules: A Systematic Review
Source: Clin Otolaryngol. 2026 Aug 2;51(6):805–18. doi: 10.1111/coa.70150 (PMC13631809; doi:10.1111/coa.70150)
Supplement: Supplementary file 5 [file COA-51-805-s005.pdf]

Appendix E1 – Search Strategy

**Database searched**	**Platform**	**Years of coverage**	**Records**	**Records after duplicates removed**
Medline ALL	Ovid	1946 - Present	59	59
Embase	Embase.com	1971 - Present	137	85
Web of Science Core Collection*	Web of Knowledge	1975 - Present	81	25
Cochrane Central Register of Controlled Trials	Wiley	1992 - Present	13	8
Additional Search Engines: Google Scholar** (50 top-ranked)			50	30
**Total**			**340**	**207**

*Science Citation Index Expanded (1975-present) ; Social Sciences Citation Index (1975-present) ; Arts & Humanities Citation Index (1975-present) ; Conference Proceedings Citation Index- Science (1990-present) ; Conference Proceedings Citation Index- Social Science & Humanities (1990-present) ; Emerging Sources Citation Index (2005-present)

***Google Scholar was searched via "Publish or Perish" to download the results in EndNote.

No database limits were used.

Embase

('radiofrequency ablation'/de OR 'radiofrequency ablation device'/de OR 'catheter ablation'/exp OR (RFA OR ((radiofrequen* OR radio-frequen* OR catheter*) NEAR/3 ablat*)):ab,ti,kw) **AND** ('thyroid tumor'/exp OR 'thyroid gland'/exp OR 'thyroid surgery'/exp OR (thyroid* OR PMTC):ab,ti,kw) **AND** ('cost effectiveness analysis'/de OR cost/exp OR 'health economics'/exp OR economics/exp OR (cost OR costs OR economic*):ab,ti,kw)

Medline

(Radiofrequency Ablation/ OR Catheter Ablation/ OR (RFA OR ((radiofrequen* OR radio-frequen* OR catheter*) ADJ3 ablat*)).ab,ti,kf.) **AND** (exp Thyroid Neoplasms/ OR Thyroid Gland/ OR (thyroid* OR PMTC).ab,ti,kf.) **AND** (Cost-Effectiveness Analysis/ OR exp Economics/ OR economics.fx. OR (cost OR costs OR economic*).ab,ti,kf.)

Cochrane

((RFA OR ((radiofrequen* OR radio-frequen* OR catheter*) NEAR/3 ablat*)):ab,ti,kw) **AND** ((thyroid* OR PMTC):ab,ti,kw) **AND** ((cost OR costs OR economic*):ab,ti,kw)

Web of Science

(TS=(RFA OR ((radiofrequen* OR radio-frequen* OR catheter*) NEAR/2 ablat*))) AND (TS=(thyroid* OR PMTC)) AND (TS=(cost OR costs OR economic*))

Google Scholar

RFA|'radiofrequency ablation'|'radio frequency ablation' thyroid|PMTC cost|costs|economic
