# Supplementary material for: Cost‐Effectiveness of Radiofrequency Ablation for Benign Non‐Functioning Thyroid Nodules: A Systematic Review
Source: Clin Otolaryngol. 2026 Aug 2;51(6):805–18. doi: 10.1111/coa.70150 (PMC13631809; doi:10.1111/coa.70150)
Supplement: Supplementary file 6 [file COA-51-805-s006.pdf]

Appendix E4 - Tables of Inflation and Currency Adjustments

Overview of Inflation and Currency Adjustments for Standardizing Costs to 2021 U.S. Dollars								
**Study**	**Original currency**	**Cost Year**	**Inflation Factor (to 2021)**	**2021 Average Exchange Rate**	**Original Mean Cost (Intervention)**	**Original Mean Cost (Comparator)**	**2021 USD-Adjusted Cost (Intervention)**	**2021 USD-Adjusted Cost (Comparator)**
Yue et al. (2016)	Chinese Yuan (CNY)	2014	1.1646	6.45 CNY per USD	CNY 18,660	CNY 13,185	USD 3,369.30	USD 2,380.72
Kuo et al. (2023)	United States Dollar (USD)	2021	n.a.	n.a.	USD 16,563	USD 19,262	USD 16,563.00	USD 19,262.00
Papi et al. (2023)	Euro (EUR)	2021	n.a.	0.85 EUR per USD	EUR 2,047.53	EUR 4,167.89 (hemithyroidectomy); EUR 5,140.99 (total thyroidectomy)	USD 2,408.86	USD 2,494.54 (hemithyroidectomy); USD 3,639,36 (total thyroidectomy)
Bernardi et al. (2014)	Euro (EUR)	2014	1.0511	0.85 EUR per USD	EUR 1,661.50	EUR 4,556.30 (inpatient); EUR 4,139.40 (short-stay)	USD 2,054.50	USD 3,579.50 (inpatient); USD 3,063.99 (short-stay)
Che et al. (2015)	Chinese Yuan (CNY)	2015	1.1418	6.45 CNY per USD	CNY 15,962	CNY 16,535	USD 2,825.63	USD 2,927.07
Elhefny et al. (2021)	Egyptian Pound (EGP)	2018	1.3389	15.64 EGP per USD	EGP 24,500	EGP 19,053	USD 2,097.40	USD 1,631.09
Schalch et al. (2021)	Brazilian Real (BRL)	2020	1.1006	5.39 BRL per USD	BRL 9,661.10	BRL 23,959.69	USD 1,972.73	USD 4,892.40
Yan et al. (2021)	United States Dollar (USD)	2017	1.1051	n.a.	USD 2,262.12	USD 2,638.04	USD 2,499.86	USD 2,915.29
Ayoub et al. (2023)	United States Dollar (USD)	2020	1.0123	n.a.	USD 8,726.38	USD 19,229.75	USD 8,834.01	USD 19,466.94
Miller et al. (2023)	United States Dollar (USD)	2018	1.0837	n.a.	USD 2582–3582 (depending on probe cost)	USD 3,179.00	USD 2798.11–3881.81 (depending on probe cost)	USD 3,445.08
Yan et al. (2023)	United States Dollar (USD)	2016	1.1329	n.a.	USD 1,979.20	USD 2,208.80	USD 2,242.14	USD 2,502.25
Kuo et al. (2024)	United States Dollar (USD)	2019	1.0539	n.a.	USD 2,059.50	USD 4,621.90	USD 2,170.50	USD 4,871.01
Lee et al. (2024)	Hong Kong Dollar (HKD)	2019	1.0238	7.77 HKD per USD	HKD 29,008	HKD 73,917	USD 3,822.13	USD 9,739.41

This table summarizes the conversion of cost data from original currencies and cost years to standardized 2021 U.S. dollars (USD) across included studies. For each study, we report the original currency and cost year, the inflation factor used to adjust to 2021 in local currency, the average market exchange rate for 2021, and both the original and converted costs for the intervention and comparator groups. Inflation indices were obtained from national statistical agencies; exchange rates reflect annual averages from the World Bank.

**Annual Consumer Price Index (CPI) Values used for Inflation Adjustment to 2021**								
**Currency**	**2014**	**2015**	**2016**	**2017**	**2018**	**2019**	**2020**	**2021**
CPI United States Dollar (USD)	435.292	446.752	463.675	475.3220	484.707	498.413	518.876	525.276
CPI Italy (EUR)	99.9	100	99.9	101.3	102.5	103.2	103	105
CPI Chinese Yuan (CNY)	102	101.4	102	101.6	102.1	102.9	102.5	100.9
CPI Hong Kong Dollar (HKD)						100.9	102.7	103.3
CPI Brazilian Real (BRL)								110.6
CPI Egyptian Pound (EGP)					111.9	107.1	105.4	105.9
This table presents the annual Consumer Price Index (CPI) values used to adjust cost estimates from their original year to 2021 values, stratified by currency. The CPI reflects the average change over time in prices for goods and services in each country or region.								
